# Does Losartan reduce the severity of COVID-19 in hypertensive patients?

**DOI:** 10.1186/s12872-022-02548-2

**Published:** 2022-03-19

**Authors:** Mohammadreza Mirjalili, Moslem Taheri Soodejani, Mehdi Raadabadi, Ali Dehghani, Fateme Salemi

**Affiliations:** 1 Internal Medicine Department, Medical College, Shahid Sadoughi University of Medical Sciences, Yazd, Iran; 2 Center for Healthcare Data Modeling, Departments of biostatistics and Epidemiology, School of public health, Shahid Sadoughi University of Medical Sciences, Yazd, Iran; 3 Health Policy and Management Research Center, School of Public Health, Shahid Sadoughi University of Medical Sciences, Yazd, Iran; 4 Biostatistics and Epidemiology Department, School of Public Health, Shahid Sadoughi University of Medical Sciences, Yazd, Iran; 5 School of Medicine, Islamic Azad University of Medical Sciences, Yazd, Iran

**Keywords:** Blood pressure/hypertension, Losartan, Mortality, SARS-CoV-2

## Abstract

**Background:**

One of the global problems is to control the coronavirus epidemic, and the role of different medicines is still unknown to policymakers. This study was conducted to evaluate the effects of losartan on the mortality rate of COVID-19 in hypertensive patients.

**Methods:**

The research sample of analytical study included 1458 patients presenting to COVID-19 diagnostic centers in Yazd that were examined in the first six months of 2020. Data were analyzed using descriptive statistics as well as chi-square, Fisher’s exact test, *t* test, and logistic regression.

**Results:**

Of 1458 subjects that were studied, 280 were hypertensive of whom 179 tested positive for SARS-CoV-2 PCR. The results showed a lower chance of death by more than 5 times in hypertensive patients who used losartan (*P* = 0.003). Moreover, regarding the effect of losartan on the prevention of COVID-19 in hypertensive patients, it was found that this medicine played a protective role although this relationship was not statistically significant (*P* = 0.86).

**Conclusions:**

The results showed that losartan reduced the chance of mortality in hypertensive patients. It is recommended that the effect of losartan and other blood pressure medicines on COVID-19 patients be investigated in larger studies as well as laboratory investigations.

## Background

Today, despite advances in medical and laboratory sciences, epidemics are one of the major problems of the medical society that threatens the lives of the general population. A series of unexplained cases of pneumonia were reported in Wuhan, China, in late December 2019. The virus was temporarily named 2019 Novel Coronavirus (2019-nCoV) by the World Health Organization on January 12, 2020. On February 11, 2020, the WHO named the virus severe acute respiratory syndrome coronavirus 2 (SARS-CoV-2) and the disease caused by the virus coronavirus disease 2019 (COVID-19. The Organization considered COVID-19 a global concern on January 30, 2020.

This virus has a high infectivity and a long incubation period so that it takes 2 to 14 days for the symptoms to appear. This characteristic increases the outbreak rate and hampers prevention and control [1]. This virus is transmitted from one person to another. It can be transmitted through respiratory droplets, direct contact with secretions containing the virus, and through the mouth, nose, and eyes.

The mortality rate is extremely high in infected patients requiring mechanical ventilation (artificial respiration) due to the severe lung damage caused by coronavirus infection [2]. The results of a study conducted in Wuhan, China, showed that 28% of the patients with COVID died [3]. The mortality rate of patients with COVID in studies conducted by Shi [4] and Liu [5] was 10.5% and 6%, respectively.

Preliminary studies have indicated that people with underlying diseases are at higher risk for complications and mortality caused by COVID-19. Nearly 50% of the hospitalized patients suspected of COVID-19 have other chronic diseases, and almost 41% of the hospitalized patients with confirmed covid-19 suffer from cardiovascular or cerebrovascular disease [6]. Among patients with COVID-19, hypertension (HTN) is a significant risk factor and a common co-morbidity for acute respiratory failure, hospitalization, and mortality [7].

So far, there is no specific antiviral drug for COVID-19 and the main treatment is supportive care such as the.

Aside from ARDS and cytokine storm, COVID19 appears to be a mild disease. Nevertheless, the acute respiratory distress syndrome (ARDS) as the primary cause of significant morbidity and death in SARS-CoV2 needs further assessments [8]. Maintaining the vital signs, regulating oxygen and blood pressure, and reducing complications such as secondary infections or organ failure are the mainstay of supportive COVID-19 management [9]. According to the RECOVERY trial, after a month, dexamethasone reduced mortality in patients hospitalized with Covid-19 getting either invasive mechanical ventilation or oxygen alone at the time of randomization. However, it showed no signs of improvement in patients without respiratory support [10]. Antiviral medications such as remdesivir, and favipiravir impair viral propagation and infectivity. Similar to anti-inflammatory agents, they are associated with a variety of adverse effects and drug-drug interactions [11]. The SARS-CoV2 receptor on host cells is Angiotensin-converting enzyme 2 (ACE2), a renin-angiotensin system (RAS) component. The RAS’s systemic and local ligands and receptors control cellular growth, metabolic rate, and salt and electrolyte balance. Therefore, antihypertensive medications such as ACE inhibitors (ACEIs) and angiotensin receptor blockers (ARBs) might improve the COVID-19 outcome [7, 8, 12–14].

After replication in the upper respiratory tract, the virus rapidly spreads to the lower airways and alveoli within a week. The pathophysiology of cytokine storm and ARDS in COVID-19 is explained precisely by hyperacute activation of AngII type 1 receptor (AT1R) by a rapid surge in intracellular Ang II due to ACE2 downregulation after SARS-CoV2 showering to the lungs. When the coronaviruses entering the body, it fuses their envelopes to the membranes of host cells and affected cells by transport their genetic material. This fusion is interposed by glycosylated spike proteins on the surface of the virion interacting with proper surface receptors on the membrane of the host cell. ACE2 receptor is a known human cell-surface protein to which CoV spike proteins specifically bind [15]. The conversion of AngI to AngII, a major effector or renin-angiotensin-aldosterone system (RAS), is mediated by ACE. ACE is a protein that is highly expressed on membranes of vascular endothelial cells, predominantly in the lung tissue [16]. Most of the physiological effects associated with RAS are mediated by the interaction of AngII with the G-protein receptor associated with AT1R, which activates the physiological pathway in various systems [15].

It is suggested that the use of losartan, an AT1R blocker with antihypertensive activity, may be beneficial in the treatment of ARDS in COVID-19. This is mainly due to the selective blockade of AT1 receptors and the reduction of the compressive effect of angiotensin II. Losartan also inhibits the development of dendritic cells and the T helper 1 immunological response; ultimately, reducing the inflammatory reactions caused by angiotensin II. Therefore, losartan could potentially protect the respiratory organs against COVID-19-induced damage [13]. It is among antihypertensive medications with the lowest side effects, nevertheless, blurry vision, dyspnea, dizziness, lightheadedness on rapid positional change, tachycardia, nausea or vomiting, and abdominal pain should be noted [17]. 

Several studies have revealed that losartan has an inhibitory effect on developing liver fibrosis and prevents aortic dilation in patients with Marfan syndrome [18, 19]. Moreover, it has been shown that losartan reduces the regulation of TGF-β1 and fibrogenic molecules in cells infected with cytomegalovirus [20]. Due to the lack of evidence regarding the effect of losartan on the COVID-19 mortality and morbidity, this study was conducted to evaluate the effect of losartan on COVID-19 mortality in hypertensive patients.

## Methods

### Study design, population and setting

All subjects suspected of COVID-19 who presented to COVID-19 laboratory and diagnostic centers were included in this analytical study. The hypertensive patients were divided to COVID- 19 positive (positive PCR test) and COVID-19 negative (negative PCR test) groups. Exclusion criteria were rejection of the general consent. The study was approved by the Ethics Committee of Shahid Sadoughi University of Medical Sciences with the ID of IR.SSU.REC.1399.101, located in Yazd, Iran.

## Variables selection

The effect of losartan on the develoment and mortality of COVID-19 was the main variable and age, diabetes, cancer, liver disease, cardiovascular disease, chronic lung disease, chronic kidney disease, and chronic nervous disease were cobnsidered as confounding factors. The presence of a heart disease was confirmed by the cardiologist member of the COVID-19 treatment team in the ICU. The data collection tool was a researcher-made questionnaire based on the data available in the COVID-19 data dashboard of Shahid Sadoughi University of Medical Sciences, Yazd, Iran.

### Statistical analysis

Frequency, percentage, mean, standard deviation, and median are used to describe the data. Chi-square, Fisher’s exact test, and *t* test were used to investigate the relationship between variables. Ultimately, logistic regression was applied for model building at a significance level of 5%.

## **Results**

**Fig. 1:**
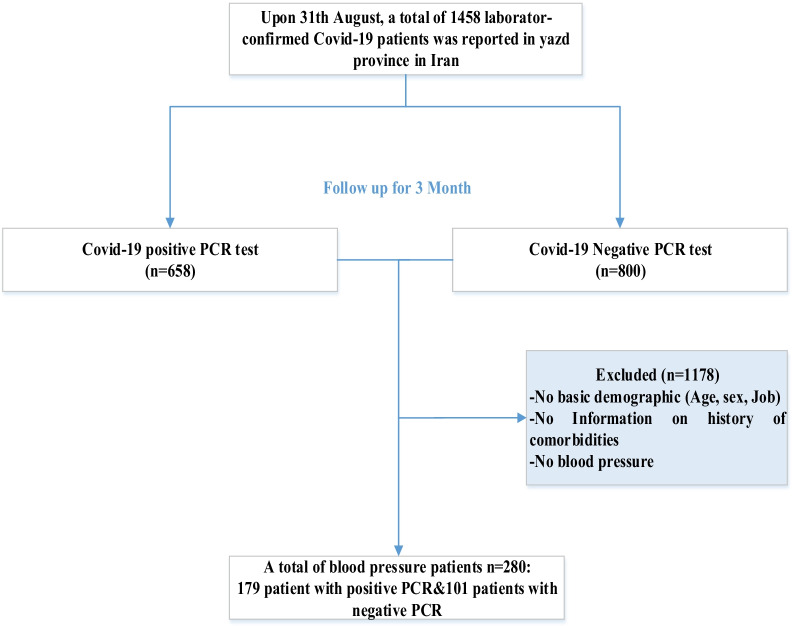
Sample flow diagram detailing included subjects and exclusion criteria

Of 1458 subjects included in this study, 658 tested positive for SARS-CoC-2 PCR and 800 tested negative. After applying the inclusion and exclusion criteria, 280 hypertensive patients were identified of whom 179 had a positive PCR test (Fig. 1]. Of 280 patients with hypertension, 134 (47.9%) were female and 146 (52.1%) were male. The mean age of the subjects was 64.60 ± 0.8 years old. Moreover, the mean, minimum and maximum age of the subjects was 66, 28, and 98 years, respectively. Table 1 provides details regarding the number of hospitalizations, respiratory failures, and ICU admissions.

**Table 1:** Characteristics of subjects with hypertension and their COVID-19 test results

Variables	Positive PCR	Negative PCR	P value
**Losartan usage**			0.054^**^
yes	59	102	
no	42	77	
**Age (Mean±SD)**	67.45±13.25	63.07±13.64	0.009^*^
**LOS (Mean±SD)**	9.02±6.40	8.14±6.27	0.285^*^
**Admission status**			0.593^**^
In-patient	176	99	
Out-patient	3	2	
**ICU admission**			0.031^**^
Yes	15	17	
No	152	79	
**Final outcome**			0.298^**^
Alive	156	91	
Dead	23	10	
**Chronic liver disease**			0.639^**^
Yes	0	1	
No	101	178	
**DM**			0.473^**^
Yes	39	77	
No	62	102	
**CVD**			0.20^**^
Yes	66	103	
No	35	76	
**CND**			0.693^**^
Yes	4	4	
No	97	175	
**Chronic Lung Disease**			0.027^**^
Yes	15	12	
No	86	167	
**CKD**			0.002^**^
Yes	15	8	
No	86	171	
**Cancer**			0.681^**^
Yes	1	1	
No	100	178	

*Abbreviations*: PCR: polymerase chain reaction, SD: standard deviation, LOS: length of stay, ICU: intensive care unit, DM: diabetes mellitus, CVD: cardiovascular diseases, CND: chronic neurological diseases, CKD: chronic kidney diseases. *Independent T test was used. **Exact test was used

Examining the effect of losartan on COVID-19 prevention in hypertensive subjects after adjustment for different variables indicated that this medicine had a non-significant protective role (P = 0.86), Table 2 presents more details. Examining the effect of losartan on the reduction of COVID-19 mortality in hypertensive patients revealed the protective role of this medicine. The results showed that patients who used this medicine had a more than 5-fold reduction in the chance of mortality, which was statistically significant (P = 0.003) (Table 3].

**Table 2:** Adjusted odds ratio for relationship between losartan usage and COVID-19 development

Factors	OR (95% CI)	*P* value
Losartan usage	0.95 (0.56, 1.61)	0.86
Age	0.97 (0.95, 0.99)	0.23
DM	1.62 (0.92, 2.84)	0.09
CVD	0.73 (0.42, 1.26)	0.256
CND	0.43 (0.10, 1.85)	0.257
Chronic Lung Diseases	0.44 (0.18, 1.02)	0.058
Kidney Diseases	0.20 (0.07, 0.55)	0.002
Cancers	0.55 (0.03, 9.29)	0.683

OR: odds ratio, CI: confidence interval, DM: diabetes mellitus, CVD: cardiovascular disorders, CND: chronic neurological diseases

**Table 3:** Adjusted odds ratio for relationship between losartan usage and COVID-19 mortality

Factors	OR (95% CI)	*P* value
Losartan usage	0.17 (0.05, 0.55)	0.003
Age	1.04 (1.00, 1.09)	0.032
DM	3.42 (1.13, 10.4)	0.030
CVD	0.44 (0.14, 1.34)	0.15
CND	1.17 (0.07, 18.54)	0.91
Chronic Lung Diseases	4.13 (0.93, 18.3)	0.063
Kidney Diseases	2.57 (0.29, 22.74)	0.394
Cancers	546 (0, Infinity)	1

OR: odds ratio, CI: confidence interval, DM: diabetes mellitus, CVD: cardiovascular disorders, CND: chronic neurological diseases

## **Discussion**

The global spread of the novel coronavirus infection has posed a serious and important threat to human health. Some studies have reported that some protease inhibitors, such as remdesivir and chloroquine, are effective in COVID-19 infection but they have not proved effective in reality [12]. Accordingly, there is now growing interest in the effect of FDA-approved medicines on COVID-19 treatment.

This study examined the effect of on the mortality rate of COVID-19 losartan in hypertensive patients. Losartan is an angiotensin II receptor antagonist that is also used to treat hypertension and reduce kidney damage in the long term in type 2 diabetic patients with hypertension [21]. According to the results of the present study, losartan has a protective role against COVID-19 mortality in patients with high blood pressure, so that the patients who used this drug had a lower chance of mortality by more than five times. Several studies have found that ACE inhibitors such as losartan may have a protective role against tissue damage. Losartan has anti-platelet aggregation and anti-diabetic properties and prevents organ damage and fibrosis [22]. Additionally, one study found that losartan prevented liver fibrosis [18], and another study reported that losartan reduced the regulation of TGF-β1 and fibrogenic molecules in cells infected with cytomegalovirus [20]. Losartan has also been recently recommended for Marfan syndrome treatment [19]. A study conducted by Salari et al. showed that losartan had a protective effect in patients with COVID-19 [12]. Another study revealed that losartan could be beneficial for COVID-19-infected patients experiencing pneumonia [23]. Nevertheless, more clinical trial studies are required in this regard.

According to the results, the COVID-19 mortality rate increased with age. In a study by Liu et al. In Hainan, China, the mortality rate was higher in the elderly versus young and middle-aged patients [5]. The results of a study conducted in the United States revealed that 80% of COVID-19 mortality occurred in patients over 65 years old [24]. These results are consistent with the results of a study conducted in China in which 80% of the mortality occurred in subjects aged 60 and over [25]. Elderly people usually develop a more severe COVID-infection and also suffer from other underlying diseases. Therefore, they require more care.

The results showed that diabetes increased the chance of death.

According to the results of other studies, patients with cardiovascular disease, diabetes, chronic respiratory disease, hypertension, and cancer have had the highest sensitivity to the disease [1, 2, 6]. In a study conducted by Zhang et al., the mortality rate was 41% higher in patients with a history of respiratory disease and 13% higher in patients with a history of heart disease [26]. I Shi et al. also found that the mortality rate was 9% higher in patients with a history of heart disease and 11% higher in patients with a history of lung disease [4]. In COVID-19 patients, pre-existing cardiovascular illness may increase the risk of myocardial damage and death [27]. Also, Nuzzi et al. reported that the occurrence of cardiac damage during hospitalization significantly raised the likelihood of poor outcome. Since most participants experiencing myocardial injury 2 days after admission had the history of hypertension, it can be concluded that optimal blood pressure regulation may lower the incidence of myocardial damage in admitted COVID-19 patients [28].

However, some studies argue that ACEIs and ARBs may enhance ACE2 receptor expression in animals, and recommend these drug classes as a COVID-19 therapeutic adjunct. Therefore, more studies regarding the mechanism of action of ARBs in the management of inflammation and cardiovascular complications in COVID-19 patients are required [13, 29]. Only 280 of the 1458 individuals studied had hypertension, with 161 of those on losartan and the rest taking other medications. Due to the small number of patients using different antihypertensive drugs, only the effect of losartan was investigated in the statistical analysis. Therefore, investigating the effects of other antihypertensive medicines in COVID-19 patients is suggested.

## Conclusions

According to the results of this study, losartan has a protective role against COVID-19 mortality in hypertensive patients. Furthermore, the mortality rate was higher in the elderly patients with underlying diseases compared to other patients. Losartan may activate intracellular defense against COVID-19 virus by producing interferon-gamma. It is recommended that the effect of losartan and other blood pressure medicines on COVID-19 patients be investigated in larger studies as well as laboratory investigations.

## Data Availability

The data-sets used and/or analyzed during the current study available from the corresponding author on reasonable request.

## Abbreviations

COVID-19: Coronavirus disease 2019
SARS-CoV-2: Severe acute respiratory syndrome coronavirus 2
PCR: Polymerase chain reaction
ACE2: Angiotensin-converting enzyme 2
Ang I: Angiotensin I
RAS: Renin–angiotensin system
